# Endoscopic removal of intrahepatic bile duct stones using a slim basket catheter passed through a device delivery system in a patient who had undergone pancreaticoduodenectomy

**DOI:** 10.1055/a-2549-3294

**Published:** 2025-03-12

**Authors:** Yuichi Hirata, Kazuhiro Iida, Kei Takahashi, Daisuke Orita, Yoshihiro Okabe

**Affiliations:** 1 469536 Gastroenterology, Kakogawa Central City Hospital, Kakogawa, Japan

Endoscopic removal of intrahepatic bile duct (IHBD) stones is often challenging in patients who have undergone pancreaticoduodenectomy 1 , especially in the right IHBD, where device insertion is complicated by the acute angle between the endoscope and the bile duct. Endoscopic removal of IHBD stones using conventional stone retrieval baskets is sometimes difficult, and reports on suitably improved devices are limited 2 . Herein, we report the case of a patient who had undergone pancreaticoduodenectomy in whom a slim basket catheter was passed through a device delivery system and used for endoscopic removal of right IHBD stones.

A 72-year-old man who had undergone pancreaticoduodenectomy for pancreatic cancer 5 years previously was admitted to our institution with acute cholangitis associated with IHBD stones ( Fig. 1 ). Endoscopic retrograde cholangiopancreatography (ERCP) performed using a colonoscope (PCF-H290TI; Olympus Medical Systems, Tokyo, Japan) revealed stones in the right IHBD. Although a helical eight-wire basket with rotational capability (RASEN2; KANEKA Medix, Osaka, Japan), a conventional stone retrieval basket (FlowerBasket V; Olympus Medical Systems), and a balloon catheter (Extractor Pro XL; Boston Scientific Japan, Tokyo, Japan) were all tried, insertion of any device into the right IHBD was difficult owing to the acute angle between the endoscope and the bile duct ( Fig. 2 ). Therefore, a tapered device delivery system (EndoSheather; Piolax Medical Device, Kanagawa, Japan) 3 was inserted into the right IHBD, allowing entry of a 5.5-Fr slim basket catheter (Memory Eight Wire Basket; Cook Medical, Bloomington, Indiana, USA). The combination of these devices achieved successful stone removal from the right IHBD ( Fig. 3 ; Video 1 ).

**Fig. 1:**
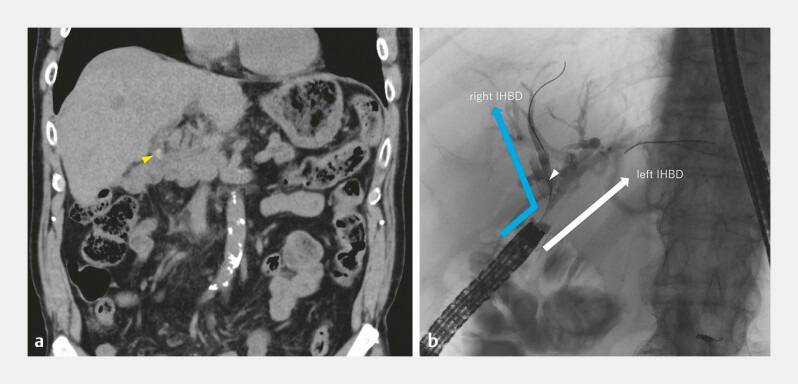
Images from a patient with acute cholangitis who had undergone previous pancreaticoduodenectomy showing on: **a** computed tomography, intrahepatic bile duct (IHBD) stones (yellow arrowhead): **b** fluoroscopy during endoscopic retrograde cholangiopancreatography, stones in the right IHBD (white arrowhead).

**Fig. 2:**
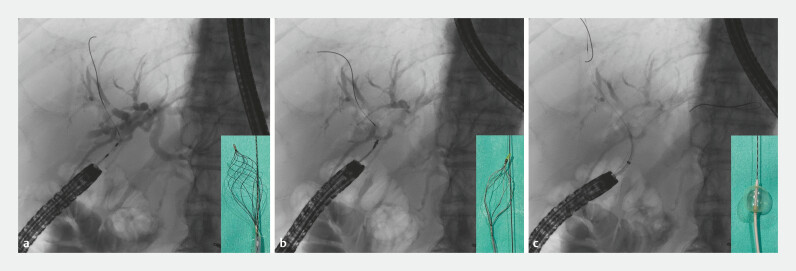
Fluoroscopic images showing the acute angle between the endoscope and the bile duct that made it difficult to insert conventional stone retrieval devices into the right intrahepatic bile duct when using the: **a** RASEN2; **b** FlowerBasket V; **c** Extractor Pro XL.

**Fig. 3:**
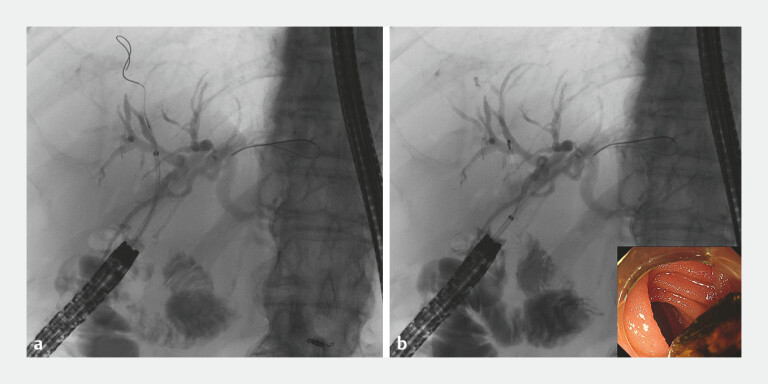
Fluoroscopic images showing: **a** the tapered device delivery system (EndoSheather) after its successful insertion into the right intrahepatic bile duct; **b** the slim basket catheter (Memory Eight Wire Basket) that was successfully passed through the device delivery system.

Endoscopic removal of intrahepatic bile duct stones using a slim basket catheter (Memory Eight Wire Basket) passed through a tapered device delivery system (EndoSheather) in a patient who had undergone pancreaticoduodenectomy.Video 1

The combination of the EndoSheather and Memory Eight Wire Basket is useful for removing IHBD stones from areas that are anatomically difficult for insertion of devices. This method may also be applicable to patients who require IHBD stone removal other than those who have previously undergone pancreaticoduodenectomy.

Endoscopy_UCTN_Code_TTT_1AR_2AH

## Notes

Endoscopy E-Videos https://eref.thieme.de/e-videos
*E-Videos* is an open access online section of the journal *Endoscopy* , reporting on interesting cases and new techniques in gastroenterological endoscopy. All papers include a high-quality video and are published with a Creative Commons CC-BY license. Endoscopy E-Videos qualify for HINARI discounts and waivers and eligibility is automatically checked during the submission process. We grant 100% waivers to articles whose corresponding authors are based in Group A countries and 50% waivers to those who are based in Group B countries as classified by Research4Life (see: https://www.research4life.org/access/eligibility/ ). This section has its own submission website at https://mc.manuscriptcentral.com/e-videos .
